# Diagnostic and Procedural Imaging Curricula in Musculoskeletal Physical Therapist Residency and Fellowship Education

**DOI:** 10.1002/pri.70218

**Published:** 2026-04-11

**Authors:** Evan Othmer Nelson, Emily Claire Goetz‐Sutinen, Tyler Alan James Amborn, Dale A. Gerke

**Affiliations:** ^1^ Department of Family Medicine and Community Health University of Wisconsin‐Madison Madison Wisconsin USA; ^2^ Department of Physical Therapy Concordia University Wisconsin Mequon Wisconsin USA

**Keywords:** education, magnetic resonance imaging, radiology, rehabilitation

## Abstract

**Background and Purpose:**

Educational standards require physical therapy (PT) orthopedic and sports residency and fellowship curricula to include diagnostic imaging instruction. Prior research found that clinicians with advanced training through post‐professional education had the requisite diagnostic imaging knowledge and skills. Curricular design or graduate proficiency in diagnostic imaging has not been investigated. The purpose of this study was to assess diagnostic and procedural imaging curricula, instructional methods, and graduate expertise in orthopedic and sports physical therapy residency and fellowship programs.

**Methods:**

A 42‐item electronic survey was distributed to all American Board of Physical Therapy Residency & Fellowship Education (ABPTRFE) orthopedic and sports programs. Content categories included (1) program characteristics, (2) diagnostic imaging curriculum, (3) instructional methods, (4) self‐reported graduate proficiency, and (5) perception that direct referral for diagnostic imaging is within the scope of PT practice.

**Results:**

Survey responses from 101 programs (84 residency; 17 fellowship) represent 67 orthopedic and 34 sports PT programs. Curricula include radiography (*n* = 90), magnetic resonance imaging (88), computed tomography (77), bone scintigraphy (55), and ultrasound (60). Eighty‐two (97%) curricula instruct how to integrate imaging results with other clinical data and 80 (94%) how to incorporate imaging results into clinical decisions. Twenty‐five (29.4%) taught how to directly refer patients for imaging studies. Curricula include diverse educational methods, resources, and assessment techniques. Programs perceived graduates were proficient in all requisite diagnostic imaging skills, but the lowest perceived proficiency was referring to imaging studies.

**Discussion:**

Imaging curricula are widely included in post‐professional orthopedic and sports PT programs. Learning experiences embedded in the clinical setting are well positioned to provide meaningful training for appropriate imaging utilization.

## 1 Introduction

Physical therapists (PTs) have the responsibility to identify time‐sensitive conditions requiring care outside their personal or professional scope of practice (American Physical Therapy Association 2023). While an ever‐present professional responsibility, the duty to refer is paramount when patients or clients access physical therapy services without referral (i.e., direct access) (American Physical Therapy Association 2020). PT authority to directly refer for diagnostic imaging is necessary to efficiently determine when physical therapy care is, or is not, appropriate. Multiple United States (US) jurisdictions allow PTs to directly refer patients for diagnostic imaging (C. Hazle et al. 2016; Mabry et al. 2020). It is imperative that clinicians are sufficiently educated in appropriate diagnostic imaging utilization. PTs with advanced training in medical imaging have demonstrated appropriate and accurate use of diagnostic imaging modalities (Crowell et al. 2016; J. H. Moore et al. 2005). Historically, the evidence base from which these conclusions were drawn represented a relatively limited number of unique practice settings (Boyles et al. 2011). More recently, research including the breadth of practice settings found that PTs frequently demonstrate diagnostic imaging knowledge, skills, and behaviors when recommending or referring for diagnostic imaging in routine practice (Rundell et al. 2021; Mabry et al. 2022; A. Keil et al. 2025). In these studies, PTs who received advanced training through residency or fellowship education were more likely to have the requisite diagnostic imaging knowledge and skills (Mabry et al. 2022; Rundell et al. 2021).

Accreditation standards require diagnostic imaging instruction in all entry level doctor of physical therapy (DPT) curricula (Commission on Accreditation in Physical Therapy Education 2023). C. R. Hazle et al. (2024) investigated the incorporation of imaging content in entry level DPT curricula. Accreditation standards also require physical therapy orthopedic and sports residency and fellowship curricula to include diagnostic imaging instruction consistent with the description of specialty practice (American Board of Physical Therapy Residency and Fellowship Education 2017; American Board of Physical Therapy Residency and Fellowship Education 2017). However, imaging curricular design and graduate proficiency in orthopedic and sports residency and fellowship programs have not been investigated. The purpose of this study was to assess diagnostic and procedural imaging curricula, instructional methods, and graduate expertise in orthopedic and sports physical therapy residency and fellowship programs.

## 2 Methods

### 2.1 Survey Development

A 42‐item survey was developed for physical therapy orthopedic and sports residencies and fellowships to evaluate the program's diagnostic imaging curriculum and graduate outcomes. Permission was obtained to adapt an established survey by Boissonnault et al. (2014) regarding imaging curricula in entry‐level DPT education. Content categories within the residency and fellowship survey included (1) program characteristics, (2) diagnostic and procedural imaging curriculum, (3) instructional methods, (4) self‐reported graduate proficiency, and (5) perception that direct referral for diagnostic imaging is within the scope of PT practice. The survey design included display logic to assure respondents were shown only relevant items. For example, if a respondent indicated that an imaging modality (e.g., ultrasound) was not included in the curriculum, subsequent survey items would not evaluate that modality. Respondents could skip any item. A partially completed survey was considered abandoned if all remaining items were unanswered. All completed items were included and skipped items were counted as no responses. Incomplete items after abandonment were excluded.

The initial version of the survey was reviewed by a PT with diagnostic imaging expertise including experience teaching diagnostic imaging in entry‐level and post‐professional settings. The recommended revisions were incorporated into the final version of the survey. A pilot test was conducted by sending the revised survey to the same PT expert and 3 PTs who teach diagnostic imaging in post‐professional physical therapist education programs. Pilot test participants reported that the survey was comprehensive and comprehensible and did not recommend revision. The final survey is presented in the supplementary material. The Institutional Review Board at Concordia University Wisconsin reviewed and approved the study protocol.

### 2.2 Survey Administration

An email contact for all orthopedic or sports residency and fellowship programs was obtained from the ABPTRFE Directory of Programs. The electronic survey was distributed to all programs using the assembled contact list (Qualtrics, Provo, UT). Contacts received an email describing the study purpose and clearly articulating that the survey was composed of items evaluating the program's diagnostic imaging curriculum and graduate outcomes. The email stated that the survey should be completed by the person with the best knowledge of the program's imaging curriculum and to forward the invitation, if necessary. A reminder email was sent to all non‐responders 17 days after the initial invitation, and a final reminder was sent 34 days later to the remaining non‐responders. Survey responses were anonymous, participants received no compensation for completing the survey, there were no time limits, and participants were not required to provide an answer to any question they did not wish to answer. Descriptive statistics characterized the participating programs and the responses to all items.

## 3 Results

Survey responses were received from 101 programs including 84 (83%) residency and 17 (17%) fellowship programs. Sixty‐seven (66%) represented orthopedic physical therapy programs and 34 (34%) represented sports physical therapy programs. Ninety‐three (92%) of programs were accredited by the American Board of Physical Therapy Residency and Fellowship Education, 5 (5%) had developing program status, and 3 (3%) did not report accreditation status. There were no differences in specialty area or accreditation status by program type. Seven residency and one fellowship survey were abandoned. For residency programs, the median [range] program length was 12 [1–24] months with 2 [1–59] residents; fellowship programs were 14 [12–36] months in duration with 4 [0–45] fellows.

Table 1 provides the reported legislative authority for patients to access physical therapy services without referral (i.e., direct access), sign diagnostic imaging referrals, or perform procedural ultrasonography in physical therapy care. Patients or clients may directly access physical therapy services at 98 (97%) of the residency and fellowship facilities, but only 20 (20%) of program facilities permitted PTs to sign diagnostic imaging referrals. Legislative authority related to procedural imaging was mixed with 46 (46%) programs reporting the authority to use ultrasound imaging as an adjunct to physical therapy care, while 34 (34%) of programs reported this was not permitted. Regulatory prohibitions against diagnostic or procedural imaging appear to hamper opportunities for physical therapy residency and fellowship programs to provide post‐professional trainees with guided practice‐based learning opportunities.

**TABLE 1:** Legislative authority for direct access or diagnostic imaging referral at orthopedic and sports residency or fellowship facilities.

	Total (*n* = 101)	Residency (*n* = 84)	Fellowship (*n* = 17)
Legislative authority for direct access			
All facilities	80 (79.2%)	68 (80.9%)	12 (70.6%)
Most or some facilities	18 (17.8%)	13 (15.5%)	5 (29.4%)
No	1 (1.0%)	1 (1.2%)	0
Unsure or unanswered	2 (2.0%)	2 (2.4%)	0
Legislative authority to sign imaging referrals			
All facilities	14 (13.9%)	10 (11.9%)	4 (23.5%)
Most or some facilities	6 (5.9%)	5 (6%)	1 (5.9%)
No	69 (68.3%)	59 (70.2%)	10 (58.8%)
Unsure or unanswered	12 (11.9%)	10 (11.9%)	2 (11.8%)
Legislative authority for procedural imaging (ultrasound as adjunct to PT treatment)			
All facilities	37 (36.6%)	29 (34.5%)	8 (47%)
Most or some facilities	9 (8.9%)	7 (8.3%)	2 (11.8%)
No	34 (33.7%)	29 (34.5%)	5 (29.4%)
Unsure or unanswered	21 (20.8%)	19 (22.6%)	2 (11.8%)

### 3.1 Diagnostic and Procedural Imaging Curricula

Imaging curricula in most residency and fellowship programs included instruction in multiple modalities (Table 2). Radiography, magnetic resonance imaging, and computed tomography are commonly included, and residency programs include imaging instruction more often than fellowship programs. While the majority of fellowship programs included instruction in imaging, 4 (24%) fellowship programs did not. One fellowship program commented that they expected imaging instruction to occur prior to fellowship education and therefore did not provide explicit imaging education. Diagnostic and procedural imaging curricula included instruction in multiple knowledge areas, skills, and abilities required of the clinical PT (Table 3). Notably, 82 (97%) curricula included instruction on how to integrate imaging results with other clinical examination data, and 80 (94%) taught how to incorporate imaging results into clinical care decisions. Only 25 (29%) provided instruction on how to directly refer patients for imaging studies.

**TABLE 2:** Diagnostic and procedural imaging modalities are included in orthopedic and sports residency and fellowship curricula.

	Total (*n* = 99)	Residency (*n* = 82)	Fellowship (*n* = 17)
Radiography (yes, *n* (%))	90 (90.9%)	77 (93.9%)	13 (76.5%)
Magnetic resonance imaging (yes, *n* (%))	88 (88.9%)	75 (91.5%)	13 (76.5%)
Computed tomography (yes, *n* (%))	77 (77.8%)	66 (80.4%)	11 (64.7%)
Bone scintigraphy (yes, *n* (%))	55 (55.6%)	47 (57.3%)	8 (47.1%)
Ultrasound (yes, *n* (%))	60 (60.6%)	51 (62.2%)	9 (52.9%)
Procedural (yes, *n* (%))	6 (6.1%)	5 (6.1%)	1 (5.9%)
Diagnostic (yes, *n* (%))	24 (24.2%)	22 (26.8%)	2 (11.7%)
Both (yes, *n* (%))	30 (30.3%)	24 (29.3%)	6 (35.3%)
None (yes, *n* (%)) (i.e., No imaging curriculum)	9 (9.1%)	5 (6.1%)	4 (23.5%)

**TABLE 3:** Diagnostic and procedural imaging instructional components are included in the orthopedic and sports residency and fellowship curricula.

Topic	Total (*n* = 85)	Residency (*n* = 72)	Fellowship (*n* = 13)
Principles and properties of imaging modalities including clinical application. (yes, *n* (%))	67 (78.8%)	58 (80.6%)	9 (69.2%)
Clinical guidelines and decision support tools that may be used to determine when to acquire imaging. (yes, *n* (%))	70 (82.3%)	60 (83.3%)	10 (76.9%)
How to recommend another provider sign an order for an imaging study. (yes, *n* (%))	49 (57.6%)	43 (59.7%)	6 (46.1%)
How to directly refer patients for imaging studies. (yes, *n* (%))^a^	25 (29.4%)	22 (30.6%)	3 (23.1%)
How to integrate imaging results with other clinical examination data during the evaluation. (yes, *n* (%))	82 (96.5%)	69 (95.8%)	13 (100%)
How to incorporate imaging results into clinical care decisions. (yes, *n* (%))^a^	80 (94.1%)	67 (93.1%)	13 (100%)
How to communicate with the radiologist or radiology department before the image is acquired. (yes, *n* (%))^a^	31 (36.5%)	23 (31.9%)	8 (61.5%)
How to communicate imaging results to patients. (yes, *n* (%))	66 (77.6%)	54 (75%)	12 (92.3%)
How to communicate imaging results with other providers after the image is acquired. (yes, *n* (%))	50 (58.8%)	39 (54.2%)	11 (84.6%)

^a^ Non‐response considered as a “no.”

### 3.2 Diagnostic and Procedural Imaging Instructional Methods

Most programs used multiple educational methods in diagnostic and procedural imaging curricula (Table 4). It was very common for didactic content to be delivered via independent reading or learning (69 programs (78%)) or lecture or classroom instruction (65 programs (73%)). Supervised patient care was an educational strategy used by 37 (42%) programs, and 53 (60%) programs included direct experience with a clinician. Most programs used multiple educational resources to support learning (Table 5); the most frequent resource was journal articles used by 75 (88%) programs. Resident or fellow proficiency in diagnostic and procedural imaging was assessed using a wide array of assessment strategies (Table 6). Written examination was the most common assessment method, assessments during routine patient care were the second most common, and case reports were the third most common.

**TABLE 4:** Diagnostic and procedural imaging instructional methods used in the orthopedic and sports residency and fellowship curricula.

Instructional method^a^	Total (*n* = 89)	Residency (*n* = 76)	Fellowship (*n* = 13)
Required independent reading or learning (yes, *n* (%))	69 (77.5%)	61 (80.3%)	8 (61.5%)
Lecture or classroom instruction (yes, *n* (%))	65 (73%)	55 (72.4%)	10 (76.9%)
Direct experience with a provider (yes, *n* (%))	53 (59.5%)	44 (57.9%)	9 (69.2%)
Online coursework (yes, *n* (%))	49 (55.1%)	45 (59.2%)	4 (30.8%)
Laboratory or active learning session (yes, *n* (%))	44 (49.4%)	38 (50%)	6 (46.1%)
Supervised actual patient care experiences (yes, *n* (%))	37 (41.6%)	28 (36.4%)	9 (69.2%)
Grand rounds or joint conferences (yes, *n* (%))	36 (40.4%)	29 (38.2%)	7 (53.8%)
Simulated patient experiences (yes, *n* (%))	29 (32.6%)	25 (32.9%)	4 (30.8%)
Recorded lecture or video coursework (yes, *n* (%))	20 (22.5%)	18 (23.7%)	2 (15.4%)
Continuing education course (yes, *n* (%))	15 (16.8%)	15 (19.7%)	0
Interactive webinar (yes, *n* (%))	2 (2.2%)	1 (1.3%)	1 (7.7%)
Other (yes, *n* (%))	1 (1.1%)	1 (1.3%)	0

^a^Non‐response considered as a “no.”

**TABLE 5:** Diagnostic and procedural imaging instructional resources used in the orthopedic and sports residency and fellowship curricula.

Educational resource^a^	Total (*n* = 85)	Residency (*n* = 72)	Fellowship (*n* = 13)
Journal articles (yes, *n* (%))	75 (88.2%)	65 (90.3%)	10 (76.9%)
Publicly available websites (yes, *n* (%))	56 (65.9%)	46 (63.9%)	10 (76.9%)
Academy of orthopedic physical therapy monographs (yes, *n* (%))	50 (58.8%)	44 (61.1%)	6 (46.1%)
Textbooks (yes, *n* (%))	41 (48.2%)	33 (45.8%)	8 (61.5%)
Imaging manuals or materials prepared by your program or health care organization (yes, *n* (%))	35 (41.2%)	30 (41.7%)	5 (38.5%)
Videos (yes, *n* (%))	25 (29.4%)	24 (33.3%)	1 (7.7%)
Published imaging manuals (yes, *n* (%))	21 (24.7%)	19 (26.4%)	3 (23.1%)
Other resources (yes, *n* (%))	4 (4.7%)	4 (5.6%)	0

^a^Non‐response considered as a “no.”

**TABLE 6:** Diagnostic and procedural imaging assessment methods used in the orthopedic and sports residency and fellowship curricula.

Assessment method	Total (*n* = 82)	Residency (*n* = 70)	Fellowship (*n* = 12)
Written examination (yes, *n* (%))	60 (73.1%)	53 (75.7%)	7 (58.3%)
Live patient examination/treatment (yes, *n* (%))	35 (42.7%)	28 (40%)	7 (58.3%)
Case report (yes, *n* (%))	21 (25.6%)	19 (27.1%)	2 (16.7%)
Practical examination (identify normal/abnormal structures on images) (yes, *n* (%))	18 (21.9%)	16 (22.9%)	2 (16.7%)
Oral examination (yes, *n* (%))	13 (15.8%)	11 (15.7%)	2 (16.7%)
Simulated patient examination/treatment (yes, *n* (%))	9 (11.0%)	7 (10%)	2 (16.7%)
Live patient referral for imaging (yes, *n* (%))	8 (9.8%)	6 (8.6%)	2 (16.7%)
Other (yes, *n* (%))	5 (6.1%)	4 (5.7%)	1 (8.3%)
Proficiency is not formally assessed (yes, *n* (%))	16 (19.5%)	13 (18.6%)	3 (25%)

### 3.3 Diagnostic and Procedural Imaging Faculty

Programs used core faculty (42 (51%)), guest faculty (17 (21%)), or a combination (24 (29%)) to deliver diagnostic and procedural imaging curricula. Instructor credentials represented multiple health care disciplines. PTs were instructors in 74 (89%) programs, 20 (24%) reported radiologist instructors, 34 (41%) non‐radiologist physicians, 7 (8%) athletic trainers, 5 (6%) physician assistants or nurse practitioners, and 4 (5%) other instructor credentials. Residency and fellowship programs had similar distributions of instructor credentials except fellowship programs did not have athletic trainers, physician assistants, or nurse practitioners. 40 (48%) of programs reported more than one instructor teaching the diagnostic and procedural imaging curriculum.

### 3.4 Diagnostic and Procedural Imaging Curriculum Outcomes

Perceived learner proficiency for multiple imaging‐specific clinical skills was reported (Supporting Information S1: Table 1). Programs perceived that the typical graduate was proficient in nearly all skills. Low perceived proficiency was reported for signing referrals for imaging studies (16%–26% proficient) and performing procedural ultrasound (27% proficient). These perceived learning outcomes are contradictory to the majority of responses supporting the expectation that residency and fellowship program graduates should have the knowledge and skills to be the ordering clinician and refer patients directly to radiologists or the radiology department (Supporting Information S1: Table 2).

## 4 Discussion

Orthopedic and sports physical therapy residency and fellowship programs leverage clinical care integration when providing diagnostic and procedural imaging curricula. Radiography and magnetic resonance imaging are the most common medical imaging modalities in musculoskeletal medicine, and it should come as no surprise that most physical therapy orthopedic and sports residencies and fellowship curricula include these imaging modalities (Ciarrapico et al. 2017). Computed tomography, bone scintigraphy, diagnostic and procedural ultrasound are included in post‐professional physical therapy curricula with a frequency mirroring the overall utilization in medicine and physical therapy practice (Ciarrapico et al. 2017; Smith‐Bindman et al. 2019; Rundell et al. 2021). All programs used a combination of educational strategies, and while lecture and independent readings were the most common educational methods, a predominant curricular strategy did not emerge. Diagnostic and procedural imaging curricula delivered by residency and fellowship programs are commonly used instructional methods that are integrated with clinical experiences (e.g., direct experience with providers). PTs were the most common instructors, but the instructor specialties reflect the interdisciplinary health care team.

The finding that some programs did not have an imaging curriculum was unexpected, and the proportion was larger in fellowship programs. Jurisdictional, organizational, or payer (e.g., Medicare) restrictions prohibiting PTs from directly referring for diagnostic imaging studies were reasons some programs chose not to include an imaging curriculum. Imaging education may not be an educational priority if imaging is viewed solely as a diagnostic tool to identify situations when physical therapy care is contra‐indicated or inappropriate. Post‐professional training, especially fellowship training, aims to advance clinical decision‐making and specialist practice. Programs in this study that included an imaging curriculum reported high perceived proficiency of residency and fellowship graduates to integrate imaging results into patient management decisions. Imaging study results may influence clinician decision‐making regarding physical therapy interventions or prognosis (Tuna et al. 2022). Therefore, post‐professional programs that include imaging education may provide more robust training than peer programs that do not.

Mabry et al. (2022) identified nine requisite skills for PTs to make imaging referrals. Physical therapy orthopedic and sports residency and fellowship curricula commonly provided instruction in the requisite clinical skills, including how to use evidence‐based imaging guidance resources, select an imaging modality, integrate imaging results into decision‐making, and communicate results to patients. The current findings complement those from Mabry et al. (2022) which demonstrate PTs with post‐professional education are more likely to use skills necessary to refer patients to a radiologist. Even though PTs frequently make imaging recommendations in clinical practice, education in how to make imaging recommendations, sign imaging referrals, and communicate with the radiology department or other clinicians were taught less frequently (Rundell et al. 2021). These skills are key components to have sufficient process knowledge to successfully make imaging referrals. The proportion of programs that provided explicit instruction in these skills aligns with the percentage of facilities with legislative authority for PTs to sign imaging referrals. Hospital‐based systems advocated for PTs to have the authority to sign imaging referrals as a component of direct access; however, analyses from routine practice show that PTs sign appropriate imaging referrals for direct access and referred patients (A. P. Keil et al. 2019). In musculoskeletal care, PTs make appropriate diagnostic imaging referrals that are necessary to determine optimal patient management (A. Keil et al. 2025). The gap between knowing when to order an appropriate imaging study and knowing how to obtain the imaging study highlights an opportunity for post‐professional training programs to better prepare graduates to practice to the fullest extent of their professional scope in any jurisdiction.

Nearly all programs indicated that their jurisdictional scope of practice allowed direct access to physical therapy services. However, a stark contrast exists in the number of programs with the authority to directly refer patients for diagnostic imaging studies. Clinician unawareness of jurisdictional regulations is a known professional issue that likely reduces opportunities for post‐professional trainees to benefit from mentored education in the clinical environment; however, the direct effect of missed opportunities cannot be determined (Rundell et al. 2021; Mabry et al. 2023). Integrating post‐professional imaging education within the clinical environment reflects a substantial but appropriate change from entry‐level physical therapy education (Hazle et al. 2024). Other health professionals recommend that education address common clinician‐specific barriers including lack of confidence when making diagnostic imaging clinical decisions or guideline non‐adherence (Perez and Jarvik 2012; Dmytriw et al. 2015; Graves et al. 2014). Post‐professional residency and fellowship curricula are well positioned to provide meaningful training for appropriate imaging utilization (DiGiovanni et al. 2014; Dolatabadi et al. 2018). As progressively more states and jurisdictions permit PTs to directly refer patients for diagnostic imaging or perform procedural imaging, it is important for clinicians at physical therapy residency and fellowship training sites to integrate diagnostic and procedural imaging into their personal scope of practice. Practice integration will create an environment where trainees are more likely to have direct learning experiences in diagnostic or procedural imaging, indirectly observe clinicians using such authority during clinical care, or be mentored by knowledgeable and experienced colleagues.

Residency and fellowship programs accreditation standards expect that “The curriculum enhances the participant's knowledge, skills, and affective behaviors through the integration of didactic instruction, focused practice, and application of evidence‐based practice principles” (American Board of Physical Therapy Residency and Fellowship Education 2023). Curriculum design provides opportunities for learning to occur, yet it is individualized based on program characteristics, resource prioritization, and contextual factors (J. G. Moore et al. 2024). Peterson et al. (2024) found that integrating post‐professional trainees into the specialty area community of practice was critical because this is where the trainees learn to adapt to the “social and context factors that influence PT care.” “While residents focus on growing as professionals to help at the patient level, residency education directly influences the organization on a grander scale, supported by a community of practice” (Peterson et al. 2024). Program leadership and faculty, representing the broad community of practice, are well positioned to guide learning that fosters the adaptive thinking required to navigate structural tensions between patient needs, jurisdictional or organizational authority, and professional responsibilities. Structured teaching and learning opportunities outside of clinical care may achieve this goal when PT referral for diagnostic imaging or performance of procedural imaging is not permitted by an organization or jurisdiction.

Responses to this survey were voluntary and anonymous. Survey responses from fellowship programs were limited in comparison to residency programs. Additionally, responses were more robust for orthopedic residency and fellowship programs compared with sports residency and fellowship programs. Another study limitation was related to the assessment of residency and fellowship graduates. There is no standardized test to assess residency and fellowship graduate proficiency regarding the use of diagnostic and procedural imaging. The qualifications for graduate proficiency with diagnostic and procedural imaging were determined independently by each respondent. Therefore, there may be variability in the level of proficiency among all residencies and fellowship graduates. Furthermore, quantifying instructional methods for diagnostic and procedural imaging was also determined independently by each respondent. Respondents frequently reported that imaging education occurred during clinical mentoring. The clinical educational setting may challenge precise determination of instructional methods and assessment techniques.

There is no standardized assessment for post‐professional imaging competence that applies to physical therapy practice. The Burley Readiness Examination is a reliable, 140‐question of musculoskeletal imaging competence, but the validity of this assessment has not been established (Burley et al. 2024). Regardless, written examinations effectively measure cognitive knowledge but are challenged to assess individualized contextual factors that are inherent to patient care. Competency‐based assessment may be a pragmatic method to assess graduate proficiency related to diagnostic or procedural imaging. While not specific to imaging, the APTA Resident Competency Evaluation Instrument provides a validated method to assess Domains of Competence that may reflect the clinical knowledge, skills, and abilities that specialist physical therapists should demonstrate (Harrington et al. 2022). Future work may identify how these emerging assessments may be used to determine imaging proficiency.

## 5 Implications for Physiotherapy Practice

Accreditation standards require orthopedic and sports physical therapy residencies and fellowship programs to include imaging education. This study is significant because it is the first report of diagnostic and procedural imaging curricula, instructional methods, and perceived graduate proficiency in orthopedic and sports physical therapy residency and fellowship programs. The findings from this study indicated that imaging curricula are widely included in physical therapy orthopedic and sports residency and fellowship programs with learning experiences embedded in the clinical setting. Survey responses reported high levels of perceived graduate proficiency in many of the requisite diagnostic imaging skills. Relatively few programs included instruction in how to directly refer patients for imaging studies and perceived graduates to have low proficiency in this skill. Nevertheless, respondents generally agreed that residency and fellowship graduates should be the referring clinician for radiographs and advanced imaging. If the clinical environment does not provide sufficient practice‐based learning opportunities, the curriculum design should provide structured learning opportunities to achieve the requisite knowledge, skills and abilities.

As additional jurisdictions allow PTs to directly refer patients for imaging studies, it is imperative that residency and fellowship programs continue to provide instruction and training in direct referral to radiologists for imaging and assess proficiency. Studies consistently demonstrate that PTs, particularly those who complete residency or fellowship training, make appropriate imaging decisions (A. Keil et al. 2025; Mabry et al. 2022; Burley et al. 2024; Nelson et al. 2022). When the need for imaging arises in routine physical therapy care, it is in the best interest of the patient, healthcare organization, payer, and clinical work system for the appropriate image to be obtained in the most efficient manner. In referred or direct access situations, the PT may be the clinician who first identifies the need for imaging. Clinical implementation of diagnostic or procedural imaging in physical therapy practice is incumbent upon clear communication with the patient and interprofessional collaboration with the radiology department or other clinical disciplines relevant to the situation (A. P. Keil et al. 2021).

## Funding

The authors have nothing to report.

## Ethics Statement

Institutional review board or ethics committee approval of study protocol: Concordia University Wisconsin.

## Conflicts of Interest

The authors declare no conflicts of interest.

## Supporting information


Supporting Information S1

## Data Availability

The data that support the findings of this study are available from the corresponding author upon reasonable request.
