# Supplementary material for: Diagnostic and Procedural Imaging Curricula in Musculoskeletal Physical Therapist Residency and Fellowship Education
Source: Physiother Res Int. 2026 Apr 11;31(2):e70218. doi: 10.1002/pri.70218 (PMC13069987; doi:10.1002/pri.70218)
Supplement: Supplementary file 1 — Supporting Information S1 [file PRI-31-e70218-s001.docx]

SUPPLEMENT

**Table S1.** Reported learner proficiency in key diagnostic imaging clinical functions upon program completion.

|  | Total | Residency | Fellowship |
| --- | --- | --- | --- |
| **Ability of a typical program graduate to identify normal anatomy** | | | |
| Radiography | n=61 | n=52 | n=9 |
| Extremely or Somewhat Proficient, n(%)  Neutral, n(%)  Somewhat or Extremely Incompetent, n(%) | 57 (93.4%)  3 (4.9%)  1 (1.6%) | 48 (92.3%)  3 (5.8%)  1 (1.9%) | 9 (100%)  0  0 |
| Magnetic Resonance Imaging | n=61 | n=52 | n=9 |
| Extremely or Somewhat Proficient, n(%)  Neutral, n(%)  Somewhat or Extremely Incompetent, n(%) | 52 (85.3%)  6 (9.8%)  3 (4.9%) | 43 (82.7%)  6 (11.5%)  3 (5.8%) | 9 (100%)  0  0 |
| Computed Tomography | n=56 | n=48 | n=8 |
| Extremely or Somewhat Proficient, n(%)  Neutral, n(%)  Somewhat or Extremely Incompetent, n(%) | 40 (71.4%)  12 (21.4%)  4 (7.1%) | 33 (68.8%)  11 (22.9%)  4 (8.3%) | 7 (87.5%)  1 (12.5%)  0 |
| Ultrasound | n=43 | n=37 | n=6 |
| Extremely or Somewhat Proficient, n(%)  Neutral, n(%)  Somewhat or Extremely Incompetent, n(%) | 18 (41.9%)  14 (32.5%)  11 (25.6%) | 16 (43.2%)  12 (32.4%)  9 (24.3%) | 2 (33.3%)  2 (33.3%)  2 (33.3%) |
| **Ability of a typical graduate to identify common pathologies or injuries** | | | |
| Radiography | n=61 | n=52 | n=9 |
| Extremely or Somewhat Proficient, n(%)  Neutral, n(%)  Somewhat or Extremely Incompetent, n(%) | 52 (85.2%)  8 (13.1%)  1 (1.6%) | 43 (82.7%)  8 (15.4%)  1 (1.9%) | 9 (100%)  0  0 |
| Magnetic Resonance Imaging | n=61 | n=52 | n=9 |
| Extremely or Somewhat Proficient, n(%)  Neutral, n(%)  Somewhat or Extremely Incompetent, n(%) | 45 (73.8%)  12 (19.7%)  4 (6.6%) | 38 (73.1%)  11 (21.2%)  3 (5.8%) | 7 (77.8%)  1 (11.1%)  1 (11.1%) |
| Computed Tomography | n=56 | n=48 | n=8 |
| Extremely or Somewhat Proficient, n(%)  Neutral, n(%)  Somewhat or Extremely Incompetent, n(%) | 35 (62.5%)  15 (26.8%)  6 (10.7%) | 29 (60.4%)  13 (27.1%)  6 (12.5%) | 6 (75%)  2 (25%)  0 |
| Ultrasound | n=43 | n=37 | n=6 |
| Extremely or Somewhat Proficient, n(%)  Neutral, n(%)  Somewhat or Extremely Incompetent, n(%) | 16 (37.2%)  15 (34.9%)  12 (27.9%) | 15 (40.5%)  12 (32.4%)  10 (27%) | 1 (16.7%)  3 (50%)  2 (33.3%) |
| **Ability of a typical graduate to recommend imaging be ordered by another provider** | | | |
| Radiography | n=61 | n=52 | n=9 |
| Extremely or Somewhat Proficient, n(%)  Neutral, n(%)  Somewhat or Extremely Incompetent, n(%) | 52 (85.2%)  8 (13.1%)  1 (1.6%) | 43 (82.7%)  8 (15.4%)  1 (1.9%) | 9 (100%)  0  0 |
| Magnetic Resonance Imaging | n=61 | n=52 | n=9 |
| Extremely or Somewhat Proficient, n(%)  Neutral, n(%)  Somewhat or Extremely Incompetent, n(%) | 47 (77.0%)  10 (16.4%)  4 (6.6%) | 38 (73.1%)  10 (19.2%)  4 (7.7%) | 9 (100%)  0  0 |
| Computed Tomography | n=56 | n=48 | n=8 |
| Extremely or Somewhat Proficient, n(%)  Neutral, n(%)  Somewhat or Extremely Incompetent, n(%) | 35 (62.5%)  15 (26.8%)  6 (10.7%) | 28 (58.3%)  14 (29.2%)  6 (12.5%) | 7 (87.5%)  1 (12.5%)  0 |
| Ultrasound | n=43 | n=37 | n=6 |
| Extremely or Somewhat Proficient, n(%)  Neutral, n(%)  Somewhat or Extremely Incompetent, n(%) | 19 (44.2%)  16 (37.2%)  8 (18.6%) | 16 (43.2%)  13 (35.1%)  8 (21.6%) | 3 (50%)  3 (50%)  0 |
| **Ability of a typical graduate to sign a referral for imaging** | | | |
| Radiography | n=61 | n=52 | n=9 |
| Extremely or Somewhat Proficient, n(%)  Neutral, n(%)  Somewhat or Extremely Incompetent, n(%) | 16 (26.2%)  19 (31.1%)  26 (42.6%) | 12 (23.1%)  15 (28.8%)  25 (48.1%) | 4 (44.4%)  4 (44.4%)  1 (11.1%) |
| Magnetic Resonance Imaging | n=61 | n=52 | n=9 |
| Extremely or Somewhat Proficient, n(%)  Neutral, n(%)  Somewhat or Extremely Incompetent, n(%) | 10 (16.4%)  23 (37.7%)  28 (45.9%) | 7 (13.5%)  19 (36.5%)  26 (50%) | 3 (33.3%)  4 (44.4%)  2 (22.2%) |
| Computed Tomography | n=56 | n=48 | n=8 |
| Extremely or Somewhat Proficient, n(%)  Neutral, n(%)  Somewhat or Extremely Incompetent, n(%) | 10 (17.9%)  21 (37.5%)  25 (44.6%) | 7 (14.6%)  18 (37.5%)  23 (47.9%) | 3 (37.5%)  3 (37.5%)  2 (25%) |
| Ultrasound | n=42 | n=36 | n=6 |
| Extremely or Somewhat Proficient, n(%)  Neutral, n(%)  Somewhat or Extremely Incompetent, n(%) | 9 (21.4%)  14 (33.3%)  19 (45.2%) | 7 (19.4%)  11 (30.6%)  18 (50%) | 2 (33.3%)  3 (50%)  1 (16.7%) |
| **Ability of a typical graduate to integrate imaging results into clinical patient management decisions** | | | |
| Radiography | n=61 | n=52 | n=9 |
| Extremely or Somewhat Proficient, n(%)  Neutral, n(%)  Somewhat or Extremely Incompetent, n(%) | 57 (93.4%)  3 (4.9%)  1 (1.6%) | 48 (92.3%)  3 (5.8%)  1 (1.9%) | 9 (100%)  0  0 |
| Magnetic Resonance Imaging | n=60 | n=51 | n=9 |
| Extremely or Somewhat Proficient, n(%)  Neutral, n(%)  Somewhat or Extremely Incompetent, n(%) | 53 (88.3%)  6 (10%)  1 (1.7%) | 46 (90.2%)  4 (7.8%)  1 (2%) | 7 (77.8%)  2 (22.2%)  0 |
| Computed Tomography | n=56 | n=48 | n=8 |
| Extremely or Somewhat Proficient, n(%)  Neutral, n(%)  Somewhat or Extremely Incompetent, n(%) | 46 (82.1%)  8 (14.3%)  2 (3.6%) | 39 (81.2%)  7 (14.6%)  2 (4.2%) | 7 (87.5%)  1 (12.5%)  0 |
| Ultrasound | n=43 | n=37 | n=6 |
| Extremely or Somewhat Proficient, n(%)  Neutral, n(%)  Somewhat or Extremely Incompetent, n(%) | 29 (67.4%)  8 (18.6%)  6 (14.0%) | 24 (64.9%)  7 (18.9%)  6 (16.2%) | 5 (83.3%)  1 (16.7%)  0 |
| **Ability of a typical graduate to communicate with other health care professionals about imaging decisions or results** | | | |
| Radiography | n=61 | n=52 | n=9 |
| Extremely or Somewhat Proficient, n(%)  Neutral, n(%)  Somewhat or Extremely Incompetent, n(%) | 50 (82.0%)  9 (14.7%)  2 (3.3%) | 41 (78.8%)  9 (17.3%)  2 (3.8%) | 9 (100%)  0  0 |
| Magnetic Resonance Imaging | n=61 | n=52 | n=9 |
| Extremely or Somewhat Proficient, n(%)  Neutral, n(%)  Somewhat or Extremely Incompetent, n(%) | 48 (78.7%)  10 (16.4%)  3 (4.9%) | 40 (76.9%)  9 (17.3%)  3 (5.8%) | 8 (88.9%)  1 (11.1%)  0 |
| Computed Tomography | n=56 | n=48 | n=8 |
| Extremely or Somewhat Proficient, n(%)  Neutral, n(%)  Somewhat or Extremely Incompetent, n(%) | 39 (69.6%)  13 (23.2%)  4 (7.1%) | 32 (66.7%)  12 (25%)  4 (8.3%) | 7 (87.5%)  1 (12.5%)  0 |
| Ultrasound | n=42 | n=36 | n=6 |
| Extremely or Somewhat Proficient, n(%)  Neutral, n(%)  Somewhat or Extremely Incompetent, n(%) | 22 (52.4%)  14 (33.3%)  6 (14.3%) | 17 (47.2%)  13 (36.1%)  6 (16.7%) | 5 (83.3%)  1 (16.7%)  0 |
| **Ability of a typical graduate to communicate imaging results or interpretations to patients** | | | |
| Radiography | n=61 | n=52 | n=9 |
| Extremely or Somewhat Proficient, n(%)  Neutral, n(%)  Somewhat or Extremely Incompetent, n(%) | 51 (83.6%)  8 (13.1%)  2 (3.3%) | 43 (82.7%)  7 (13.5%)  2 (3.8%) | 8 (88.9%)  1 (11.1%)  0 |
| Magnetic Resonance Imaging | n=61 | n=52 | n=9 |
| Extremely or Somewhat Proficient, n(%)  Neutral, n(%)  Somewhat or Extremely Incompetent, n(%) | 50 (82.0%)  7 (11.5%)  4 (6.6%) | 42 (80.8%)  6 (11.5%)  4 (7.7%) | 8 (88.9%)  1 (11.1%)  0 |
| Computed Tomography | n=56 | n=48 | n=8 |
| Extremely or Somewhat Proficient, n(%)  Neutral, n(%)  Somewhat or Extremely Incompetent, n(%) | 41 (73.2%)  10 (17.9%)  5 (8.9%) | 34 (70.8%)  9 (18.8%)  5 (10.4%) | 7 (87.5%)  1 (12.5%)  0 |
| Ultrasound | n=43 | n=37 | n=6 |
| Extremely or Somewhat Proficient, n(%)  Neutral, n(%)  Somewhat or Extremely Incompetent, n(%) | 25 (58.1%)  10 (23.2%)  8 (18.6%) | 21 (56.8%)  8 (21.6%)  8 (21.6%) | 4 (66.7%)  2 (33.3%)  0 |
| **Perform procedural ultrasonography as a technique during PT interventions** | | | |
| Ultrasound | n=26 | n=21 | n=5 |
| Extremely or Somewhat Proficient, n(%)  Neutral, n(%)  Somewhat or Extremely Incompetent, n(%) | 7 (26.9%)  5 (19.2%)  14 (53.8%) | 6 (28.6%)  4 (19.0%)  11 (52.4%) | 1 (20%)  1 (20%)  3 (60%) |
| *Note: Total responses include only obtained responses. Missing or incomplete responses were not included. Branch logic: only shown to programs including imaging instruction on the specific modality AND reported assessment of proficiency.* | | | |

**Table S2.** Expectations for PT Residency and Fellowship Graduates to Refer for Imaging Studies by Modality.

| Question: For each type of imaging modality, do you feel graduates of physical therapy residency and fellowship programs should have the knowledge and skills to be the ordering provider and refer patients directly to radiologists/radiology departments? | | | |
| --- | --- | --- | --- |
|  | Total | Residency | Fellowship |
| Radiography | n=77 | n=65 | n=12 |
| Strongly agree, n(%)  Agree, n(%)  Somewhat agree, n(%)  Neither agree nor disagree, n(%)  Disagree*, n(%) | 44 (57.1%)  21 (27.2%)  7 (9.1%)  4 (5.2%)  1 (1.3%) | 38 (58.5%)  16 (24.6%)  7 (10.8%)  3 (4.5%)  1 (1.5%) | 6 (50%)  5 (41.7%)  0  1 (8.3%)  0 |
| Magnetic Resonance Imaging | n=76 | n=64 | n=12 |
| Strongly agree, n(%)  Agree, n(%)  Somewhat agree, n(%)  Neither agree nor disagree, n(%)  Disagree*, n(%) | 30 (39.5%)  21 (27.6%)  13 (17.1%)  9 (11.8%)  3 (10%) | 25 (39.1%)  15 (23.4%)  13 (20.3%)  8 (12.5%)  3 (4.7%) | 5 (41.7%)  6 (50%)  0  1 (8.3%)  0 |
| Computed Tomography | n=76 | n=65 | n=11 |
| Strongly agree, n(%)  Agree, n(%)  Somewhat agree, n(%)  Neither agree nor disagree, n(%)  Disagree*, n(%) | 23 (30.3%)  16 (21.0%)  20 (26.3%)  13 (17.1%)  4 (5.3%) | 19 (29.2%)  11 (16.9%)  20 (30.8%)  11 (16.9%)  4 (6.2%) | 4 (36.4%)  5 (45.5%)  0  2 (18.2%)  0 |
| Ultrasound | n=77 | n=65 | n=12 |
| Strongly agree, n(%)  Agree, n(%)  Somewhat agree, n(%)  Neither agree nor disagree, n(%)  Disagree*, n(%) | 29 (37.7%)  24 (31.1%)  15 (19.5%)  7 (9.1%)  2 (2.6%) | 23 (35.4%)  19 (29.2%)  15 (23.1%)  6 (9.2%)  2 (3.1%) | 6 (50%)  5 (41.7%)  0  1 (8.3%)  0 |
| Bone Scintigraphy | n=77 | n=65 | n=12 |
| Strongly agree, n(%)  Agree, n(%)  Somewhat agree, n(%)  Neither agree nor disagree, n(%)  Disagree*, n(%) | 19 (24.7%)  17 (22.1%)  20 (26.0%)  17 (22.1%)  4 (5.2%) | 16 (24.6%)  12 (18.5%)  18 (27.7%)  15 (23.1%)  4 (6.1%) | 3 (25%)  5 (41.6%)  2 (16.7%)  2 (16.7%)  0 |
| *Due to small response frequency, “Disagree” combines respondent choices of “Somewhat disagree,” “Disagree,” “Strongly disagree” | | | |
